# Escalation of care in children at high risk of clinical deterioration in a tertiary care children’s hospital using the Bedside Pediatric Early Warning System

**DOI:** 10.1186/s12887-022-03555-0

**Published:** 2022-09-07

**Authors:** Orsola Gawronski, Jos Maria Latour, Corrado Cecchetti, Angela Iula, Lucilla Ravà, Marta Luisa Ciofi degli Atti, Immacolata Dall’Oglio, Emanuela Tiozzo, Massimiliano Raponi, Christopher S. Parshuram

**Affiliations:** 1 Professional Development, Continuing Education and Research Unit, Medical Directorate, Bambino Gesù Children’s Hospital IRCCS, P.zza S. Onofrio 4, Rome, Italy; 2 Faculty of Health, School of Nursing and Midwifery, University of Plymouth, Plymouth, UK; 3 School of Nursing, Midwifery and Paramedicine, Faculty of Health Sciences, Curtin University, Perth, Australia; 4 Pediatric Intensive Care Unit, Department of Emergency, Acceptance and General Pediatrics, Bambino Gesù Children’s Hospital IRCCS, P.zza S. Onofrio 4, Rome, Italy; 5 Clinical Epidemiology Unit, Bambino Gesù Children’s Hospital IRCCS, P.zza S. Onofrio 4, Rome, Italy; 6 Medical Directorate, Bambino Gesù Children’s Hospital IRCCS, P.zza S. Onofrio 4, Rome, Italy; 7 Paediatric Intensive Care Unit, Critical Care Program, Hospital for Sick Children, 555 University Ave, Toronto, ON M5G1X8 Canada

**Keywords:** Escalation of care, Pediatric, Track and trigger tool, BedsidePEWS, PEWS, Intensive care, Urgent admission

## Abstract

**Background:**

Escalation and de-escalation are a routine part of high-quality care that should be matched with clinical needs. The aim of this study was to describe escalation of care in relation to the occurrence and timing of Pediatric Intensive Care Unit (PICU) admission in a cohort of pediatric inpatients with acute worsening of their clinical condition.

**Methods:**

A monocentric, observational cohort study was performed from January to December 2018. Eligible patients were children: 1) admitted to one of the inpatient wards other than ICU; 2) under the age of 18 years at the time of admission; 3) with two or more Bedside-Paediatric-Early-Warning-System (BedsidePEWS) scores ≥ 7 recorded at a distance of at least one hour and for a period of 4 h during admission. The main outcome -the 24-h disposition – was defined as admission to PICU within 24-h of enrolment or staying in the inpatient ward. Escalation of care was measured using an eight-point scale—the Escalation Index (EI), developed by the authors. The EI was calculated every 6 h, starting from the moment the patient was considered eligible. Analyses used multivariate quantile and logistic regression models.

**Results:**

The 228 episodes included 574 EI calculated scores. The 24-h disposition was the ward in 129 (57%) and the PICU in 99 (43%) episodes. Patients who were admitted to PICU within 24-h had higher top EI scores [median (IQR) 6 (5–7) vs 4 (3–5), *p* < 0.001]; higher initial BedsidePEWS scores [median (IQR) 10(8–13) vs. 9 (8–11), *p* = 0.02], were less likely to have a chronic disease [*n* = 62 (63%) vs. *n* = 127 (98%), *p* < 0.0001], and were rated by physicians as being at a higher risk of having a cardiac arrest (*p* = 0.01) than patients remaining on the ward. The EI increased over 24 h before urgent admission to PICU or cardiac arrest by 0.53 every 6-h interval (CI 0.37–0.70, *p* < 0.001), while it decreased by 0.25 every 6-h interval (CI -0.36–0.15, *p* < 0.001) in patients who stayed on the wards.

**Conclusion:**

Escalation of care was related to temporal changes in severity of illness, patient background and environmental factors. The EI index can improve responses to evolving critical illness.

**Supplementary Information:**

The online version contains supplementary material available at 10.1186/s12887-022-03555-0.

## Background

Late Pediatric Intensive Care Unit (PICU) admission and failure-to-rescue in children admitted to hospital wards is often a consequence of missed signs of increasing clinical deterioration, ineffective observations, low situational awareness and/or failure in the response system [1, 2]. Escalation of care has been defined as an organizational response to different levels of abnormal or physiological measurements or other forms of observed deterioration [3]. This process requires the timely identification of deterioration, communication among team members, and appropriate interventions.

**Fig. 1:**
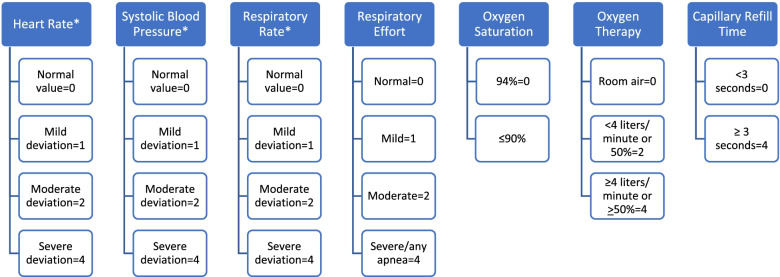
The Bedside Pediatric Early Warning System clinical indicators and subscores. *vital sign ranges are subdivided according to five age groups (0–3 months, 3–12 months, 1–4 years, 4–12 years, > 12 years). Note: adapted from Parshuram C. S. et al., 2011

Pediatric track and trigger tools provide recommendations for graduated escalation to be matched with the patient’s severity of illness, according to the early warning score or other triggers. The Bedside Paediatric Early Warning System (BedsidePEWS) is a validated pediatric score and system, which has undergone rigorous validation [4–6] and is one of the best performing screening tools for hospital wards [7]. Figure 1 shows the BedsidePEWS score clinical indicators and subscores.

We hypothesized that amongst sick children, those with initially elevated BedsidePEWS scores could: [i] get better over a 24-h period: their BedsidePEWS scores should fall, their doctors and nurses should notice that they are at lower risk of having a cardiac arrest, and their care should be de-escalated, and they should not be admitted to the PICU within 24 h or immediately afterwards; or [ii] not improve (remain stable or get worse) over a 24-h period: their BedsidePEWS scores may remain the same or increase, their doctors and nurses should notice that they are at a higher risk of cardiac arrest, and therefore care should be escalated and be admitted to PICU within 24 h.

To our knowledge, escalation practices, timing, and trends have not been described for patients at high risk of clinical deterioration with elevated BedsidePEWS scores (BedsidePEWS ≥ 7), nor a comparison between escalation practices on high-scoring children urgently admitted to PICU compared to children admitted to routine hospital wards.

Thus, the primary aim of this study was to describe the escalation of care including the occurrence and timing of PICU admission in a cohort of pediatric in-patients with acute worsening of their clinical condition. Secondary aims were [i] to identify patient characteristics associated with the escalation of care, and [ii] to compare healthcare professionals’ perceptions of the risk of clinical deterioration in children admitted to PICU vs those remaining on a hospital ward.

## Methods

A monocentric, observational cohort study was performed. The hospital Ethics Committee reviewed and approved the study protocol (EC n 915_OPBG_2015).

Eligible inpatient units were ten hospital inpatient wards, including the Cardiology Unit, General Pediatrics, two Stem Cell Transplant and Hematology-Oncology Units, four Pediatric specialty wards, the sub-intensive care Pediatric Unit and the Pediatric Emergency Care Unit. Ineligible areas were the three PICUs, the Neonatal Department and the Outpatients Services. Eligible patients were admitted to one of the participating units, were under the age of 18 years at the time of hospital admission, and had two or more documented BedsidePEWS scores ≥ 7 separated by at least one hour during a period of 4 h. Repeated enrolment was permitted in patients who were enrolled and were subsequently discharged from an ICU. Children with BedsidePEWS ≥ 7 were enrolled for this study as this cut-off score indicates a high risk of critical deterioration and a PICU consult is locally and elsewhere recommended [5].

**Fig. 2:**
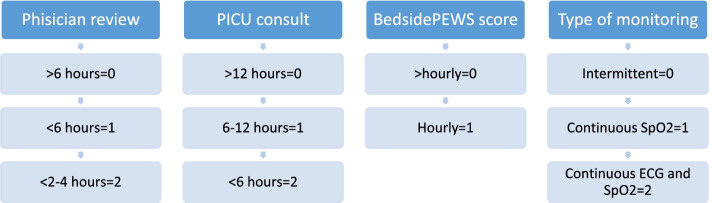
Escalation index for patients with BedsidePEWS score ≥ 7: score items and points

The main outcome was 24-h patient disposition. Patients were either in the PICU or in an inpatient ward. The main predictor of interest was escalation of care, which was measured using the Escalation Index (EI). The EI is a composite measure developed by the authors derived using the following domains: monitoring technology, vital sign frequency; and secondary consultation. These domains are aligned with the BedsidePEWS Score Matched Care Recommendations (SMCR). The EI ranges from 0 (minimum escalation) to a maximum of 7 (Fig. 2).

Secondary outcomes were the healthcare professionals’ retrospective ratings of the patients’ clinical deterioration and the intensity of care provided by healthcare professionals (HCPs) as determined by the Children’s Resuscitation Intensity Scale (CRIS) reported in supplementary file 1.

### Study context

The BedsidePEWS has been used in our hospital since 2014. The seven-item BedsidePEWS score ranges between 0 and 26. The score was reported to identify children at risk for cardiopulmonary arrest with a very good performance, reported by an AUROC curve of 0.87 (95% CI = 0.85 to 0.89). Increasing scores were reported as significantly associated to clinical deterioration events [6]. A randomized controlled cluster trial showed a significant reduction of significant clinical deterioration events in hospitals using the BedsidePEWS [5].

The BedsidePEWS score matched care recommendations (SMCRs) for escalation of care are matched with the BedsidePEWS score ranges. They have been defined according to the consensus of more than 280 healthcare professionals on reasonable care in the domains of vital signs assessment, continuous, intermittent or type of monitoring, nursing, medical and ICU review, and the number of patients per nurse according to patients’ risk by score [8]. Escalation of care of patients at high risk of clinical deterioration, set by a response system policy that includes the BedsidePEWS, involves an increasing frequency of monitoring and nursing or medical reviews including PICU consultation, in relation to patient risk. The BedsidePEWS SMCRs are not intended to substitute but to support HCPs’ clinical judgement and situational awareness of deteriorating children, which are the main drivers of escalation of care. The SMCRs for BedsidePEWS ≥ 7 are reported in Table 1.

**Table 1:** Score matched care recommendations for BedsidePEWS ≥ 7

BedsidePEWS score range	BedsidePEWS = 7–8	BedsidePEWS > 8
Vital signs documentation	15–60 min	15 min (15–60 min if stable*)
Nursing re-evaluation	2 h (4 h if stable*)	15 min
Medical evaluation	2 h (4 h if stable*)	15 min
Type of monitoring	ECG, SpO2	ECG, SpO2
PICU consult	Evaluate	Evaluate
Additional patients of same risk score/nurse	0–1 patients	0

Note* this recommendation is applied to children who remain within this risk range after the first assessment

*ECG*  electrocardiogram monitoring, *SpO2* peripheral oxygen saturation, *PICU* Pediatric Intensive Care Unit

Patients with higher acuity are cared for on step down/sub-intensive care units, where advanced treatments such as inotropes or non-invasive ventilation can be provided. The ward team is responsible for patients and consultations. PICU physicians can be consulted to see a patient in a hospital ward by a ward physician or by a ward nurse. The BedsidePEWS recommends a PICU consult when the BedsidePEWS score is ≥ 7.

### Study measures and procedures

Two researchers (OG, AI) performed daily patient screenings by consulting the electronic patient register, examining the clinical information and the BedsidePEWS scores on the medical handover records. Patients meeting the inclusion criteria had clinical data abstracted from their medical records by trained research nurses. Patient characteristics and clinical data on risk factors for cardiac arrest, BedsidePEWS scores, medical reviews, monitoring, ICU consultation and other clinical interventions were collected directly from clinical records. Data collection began when the BedsidePEWS reached the first score ≥ 7 and continued, for the following 24 h. Data on the BedsidePEWS and escalation index was grouped into 6-h blocks for 24 h after enrolment. An EI was calculated for each 6-h block according to the interventions provided in response to the first BedsidePEWS score ≥ 7 documented within that time interval. Clinical data were abstracted and entered into a database. Data were checked for consistency and accuracy by a second independent research nurse. Inconsistencies were resolved by checking the medical records and discussion among the research group.

Nurses and physicians who cared for the enrolled patients during the observation period were interviewed within 72 h of patient enrollment to provide additional data on their perception of the patient’s clinical condition and escalation of care. They completed a survey to describe their retrospective global rating of the risk of clinical deterioration and actions envisioned for that patient. They were asked ‘How much would you have been surprised if this patient arrested?’; ‘Would you have called for an urgent PICU consult for this patient?’ Responses were recorded on a 10-point Likert scale.

### Analysis

Data were described through means and standard deviations or median and interquartile ranges, as appropriate, according to the distribution, tested with D’Agostino Pearson test. Inferences were calculated with chi-square, by Student’s T-test and Mann–Whitney U Test according to the distribution. A *p* < 0.05 was considered as significant.

From these data we calculated the maximum EI within the 24 h of a PICU admission or progression of ward admission. A linear mixed effect regression model was performed to evaluate the temporal evolution of the EI preceding urgent PICU admissions. The dependent variable was the first EI calculated during the 6-h interval before the unplanned admission. The independent variable was the time interval before an unplanned PICU admission.

A quantile regression and a multivariate model was used to describe patient characteristics, the healthcare professionals’ retrospective rating of clinical deterioration and other factors associated with the escalation of care.

A Proportional Hazard Cox Regression model was performed to describe the association of the EI and other factors to PICU admission by time interval. Adjustment was performed by chronic disease, patient complexity (> 10 medications), recent transfer, isolation, CRIS, highest BedsidePEWS score, total length of stay, EI by time interval, highest EI, nurses’ and doctors’ rating of clinical deterioration and need for PICU transfer, and the nurse/patient ratio. Chronic disease was defined as an illness that lasts for 3 months or more, or that requires long term care.

Survey data from frontline nurses and physicians were paired with corresponding data from patients while on the wards in the 24-h study period, and were used to calculate the maximum EI score. When more than one physician or nurse was surveyed, the one that cared for that patient closer to the event was selected. The responses of the frontline physicians and nurses were represented on a numerical scale from 1 to 10. Adjustment was made for predictors of escalation of care: the highest BedsidePEWS score in 24 h, chronic disease, isolation, complexity (> 10 medications), recent transfer from other units or services, diagnosis, and age.

The subgroup analyses of the EI was performed for the following domains: age, chronic disease, diagnosis, reason for admission, isolation, complexity (> 10 medications), devices, recent transfer from another ward or service, BedsidePEWS score.

## Results

The study was conducted between January and December 2018 in 10 eligible inpatient units of a 607-bed tertiary care pediatric hospital. The 225 children included in this study experienced 228 episodes, and 574 6-h blocks were evaluated. The mean age was 3.53 (SD ± 5.24) years. Of the included children, 189 (83%) had a chronic disease and the most common reason for admission was respiratory disorder (*n* = 95, 42%). The median (IQR) BedsidePEWS score at enrolment was 8 (7-9). In each patient, the BedsidePEWS scores were ≥7 a median (IQR) of 5 (3-8) times in 24 h after enrolment. The 24-h disposition was the ward in 129 patient-episodes and the PICU in 99 patient-episodes. PICU admission occurred within 6 h of meeting eligibility in 37 (37%) children, in 6–12 h in 13 (13%) children, and in 12–18 h in 17 (17%) children.

### Escalation of care

EI scores for the initial 6-h period reflected children having continuous saturation and ECG monitoring (86%), vital sign assessments at a 1–4 h frequency (73%) and physician review within 4 h (62%) (Supplementary Electronic Table 1.) EI scores increased from the initial levels in children who were urgently admitted to the PICU; and were significantly higher than children who were not admitted to the PICU within 24 hours (median (IQR) of 6 (5-7) vs 4 (3-5), *p*<0.001). Escalation of care upon enrolment was significantly different in children urgently transferred to PICU, compared to ward patients in the domains of vital signs monitoring frequency and PICU consult (*P* < 0.001). When stratifying for age, respiratory, cardiovascular and oncological disease, any reason for admission, chronicity, recent transition, complexity of care (> 10 medications) and isolation, the highest EI score was significantly higher in patients with PICU urgent admission compared to patients remaining on the ward (Table 2).

**Table 2:** Escalation index in 228 patient episodes ^a^

	**PICU admission**		**Ward**		
**Patient characteristics**	**N (%)**	**Median (IQR) EI**	**N (%)**	**Median (IQR) EI**	***p* value**
All	99	6 (5–7)	129	4 (3–5)	< 0.001
Age					
1 year	43 (43)	6 (5–7)	58 (45)	4 (4–4)	< 0.001
1- < 5 years	29 (29)	5 (4–7)	40 (31)	4 (3–5)	0.001
5- < 12 years	11 (11)	7 (5–7)	10 (8)	4 (3–5)	0.004
≥ 12 years	16 (16)	6 (5–7)	32 (16)	4 (3–5)	< 0.001
Chronic disease	62 (63)	6 (4–7)	127 (98)	4 (3–5)	< 0.001
Diagnosis					
Respiratory	45 (45)	6 (5–7)	50 (39)	4 (4–5)	< 0.001
Cardiovascular	12 (12)	5.5 (4–6)	53 (41)	4 (3–4)	< 0.001
Neurological	12 (12)	5 (4–7)	12 (9)	3 (2.5–4.5)	0.062
Blood cancer	24 (24)	6 (4–7)	13 (10)	4 (3–5)	0.002
Reason for admission					
Respiratory	51 (52)	6 (5–7)	60 (47)	4 (4–5)	< 0.001
Cardiovascular	11 (11)	5 (4–6)	42 (33)	4 (3–4)	< 0.001
Abdominal	7 (7)	5 (5–7)	3 (4)	4 (3–4)	0.007
Infection	6 (6)	7 (6–7)	7 (5)	4 (2–5)	0.001
Blood cancer	14 (14)	6 (5–7)	13 (10)	5 (4–5)	0.049
Isolation	39 (39)	6 (5–7)	39 (31)	4 (4–5)	< 0.001
Medications (≥ 10)	52 (53)	6 (5–7)	78 (60)	4 (3–4)	< 0.001
Recent transition	37 (37)	6 (5–7)	31 (24)	4 (4–5)	< 0.001
Type of transition					
24 h from ER	20 (20)	7 (6–7)	18 (14)	4 (4–6)	0.002
48 h from PICU	6 (6)	5.5 (4–6)	12 (9)	4 (3.5–4)	0.075

^a^*EI* Escalation index

Data are from 228 patients with two consequent BedsidePEWS ≥ 7 within 4 h of admission on a paediatric ward. The maximum Escalation Index over 6-hourly evaluations was calculated for 24 h ending on the last 6-h interval before an urgent PICU admission or progression of ward admission. The Escalation Index was higher in patients admitted to PICU compared with all patients who remained on the ward in each category. ER = Emergency Room; PICU = Pediatric Intensive Care Unit.

A significant correlation between the BedsidePEWS and the EI was found (Spearman *r* = 0.31, *P* < 0.0001). Univariate quantile regression analyses showed an association between the independent variable, the highest EI and the highest BedsidePEWS, the absence of chronic disease, not having cardiovascular and neurological diagnosis, isolation, complexity (> 10 medications), < 24 h transition from Emergency Room or primary care service. The multivariate quantile regression showed an association of the highest EI and the maximum BedsidePEWS and isolation. An inverse association was found for chronic disease and complexity (> 10 medications) in the 24 h observation period (Table 3).

**Table 3:** Patient characteristics associated with escalation of care^a^

Characteristic	**Univariate**			**Multivariate**		
	Coeff	*p* value	95% CI	Coeff	*p* value	95% CI
**Age**						
1 year	**-**	-	-	-	-	**-**
1- < 5 years	0	1	-0.74–0.74	-0.30	0.3	-0.88–0.27
5- < 12 years	1	0.085	-0.14–2.14	-0.75	0.5	-1.27–0.57
≥ 12 years	1	0.032	0.09–1.91	-0.77	0.06	-1.56–0.02
**Chronic disease**	-2	< 0.001	-2.5—-1.45	-0.90	0.01	-1.61-—1.20
**Diagnosis**						
Respiratory	-	-	-	-	-	-
Cardiovascular	-1	0.005	-1.7—0.3	-0.63	0.09	-1.44- 0.09
Neurological	-1	0.05	-2–0.01	-0.07	0.89	-1.05–0.91
Onco-haematological	0	1	-0.84–0.84	0.72	0.15	-0.27–1.71
Other	0	1	-1.7–1.7	0.16	0.88	-1.05–1.91
**Isolation**	1	0.001	0.42–1.58	0.67	0.009	0.17–1.17
**Medical devices**	0	1	-0.64–0.64	0.32	0.27	-0.27–0.97
**Medications (≥ 10)**	-1	0.001	-1.59–0.4	-0.60	0.04	-1.19- -0.02
**Transitions**						
No transitions	-	-	-	-	-	-
24 h from ER or primary service	2	< 0.001	1.40–2.60	0.47	0.18	-0.21–1.15
48 h from PICU	0	1	-0.84–0.84	- 0.67	0.13	-1.53–0.21
48 h from other wards	1	0.07	-0.1–2.1	-0.23	0.68	-1.35–0.89
**Maximum BPEWS**	0.13	0.02	0.02–0.2	0.21	< 0.001	0.12–0.30

*BPEWS* BedsidePEWS, *ER* = Emergency Room, *PICU* Pediatric Intensive Care Unit

^a^ Data are from 228 patients. Factors associated to the Highest median Escalation Index are described through a multivariable quantile regression. Significant associations found patients with higher escalation index values had greater BedsidePEWS scores and were more often in isolation. Less escalation of care occurred in patients with chronic disease and those receiving more than 10 medications. Diagnosis, presence of medical devices, transitions of care and age were not associated with the extent of escalation in multi-variable models

**Fig. 3:**
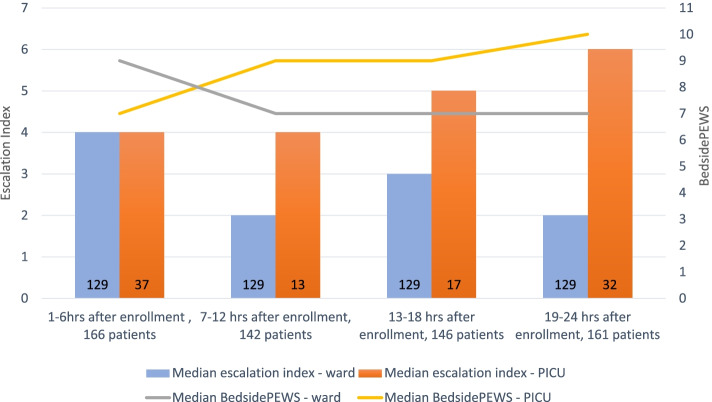
Progression of the escalation of care and the BedsidePEWS of high-risk patients (BedsidePEWS ≥7). ^a^Data are from a total of 228 patients. The graph represents the trend of the median value of the Escalation Index and the highest BedsidePEWS during the 24 h observation period in the 99 patients urgently admitted to PICU and the 129 patients who stayed on a hospital ward. Legend: PICU = Pediatric Intensive Care Unit

The EI was 1.63 times higher in children urgently admitted to PICU than in children who stayed on the wards (CI 1.29–1.97, *p* < 0.0001). A mixed effect regression model showed that out of a total of 223 episodes, the EI increased over 24 h before PICU urgent admission or cardiac arrest by 0.53 every 6-h time interval (CI 0.37–0.70, *p* < 0.001). In children who remained on the wards, over a total of 351 patient episodes, the EI decreased over the 24 h after the first BedsidePEWS ≥7 by 0.25 every 6-h interval (CI -0.36–0.15, *p* < 0.001), The median values of the EI and the highest BedsidePEWS by time interval among ward and PICU patients is presented in Fig. 3.

The Proportional Hazard Cox Regression model found the Hazard Ratio of unplanned PICU admission increased by 42% at 12–6 h and by 39% < 6 h from PICU admission for every unit increase of the escalation index. No other variables included in the model were found significant.

### Healthcare professional ratings

There were 102 physicians (45%) and 120 nurses (52%) who retrospectively rated the potential for clinical deterioration of the patient they provided care for and the ‘need’ for a PICU consult. A quantile regression adjusting for predictors of escalation of care found that HCPs’ clinical deterioration rating (physicians β coeff = 0.28, *p* = 0.003; nursing rating, β coeff = 0.16, *p* = 0.025) and HCPs’ perception of the importance of obtaining a PICU consult (physician’s rating, β coeff = 0.23, *p* < 0.001; nursing rating, β coeff = 0.11, *p* = 0.03) were significantly associated with the highest EI during the 24-h period.

Among PICU patients, the retrospective HCPs’ ratings of the patients’ clinical deterioration (52 patients, 53%) and the nurses’ ratings of their need for a PICU consult (58 patients, 59%) was also significantly associated with the EI (Clinical deterioration rating, β coeff = 0.44, CI = 0.11–0.76, *p* = 0.01; Need for PICU consult, β coeff = 0.32, CI = 0.11–0.54, *p* = 0.004). No significant association was found with the highest BedsidePEWS score.

### ICU admission

Compared with patients who remained on the ward, patients who were urgently admitted to PICU within 24 h of enrolment were less likely to have chronic health conditions of any sort or cardiovascular reasons for admission, were more likely to have respiratory or haemato-oncological conditions, a recent transfer from other wards or services (all *p* < 0.0001), had a higher maximum BedsidePEWS score [median (IQR) 10 (8-13) vs 9 (8-11), *p*=0.02], higher EI scores [6 (5-7) vs 4 (3-5), *p* < 0.0001] and related proportions of continuous monitoring, vital signs documentation, physician reviews, and PICU consultations (Table 4).

**Table 4:** Patient characteristics, by timing of PICU or ward admission

**PICU admission**	**YES**					**NO**	
**Time from enrollment, hours**	≤ 6*	**7–12**^a^	**13–18**^a^	**19–24**^a^	** < 6–24**^a^	**At 24**	*P* value **
**Patients**, n (%)	37 (37)	13 (13)	17 (17)	32 (32)	99	129	
**Age**, median (IQR), years	1 (0–5)	1 (0–5)	1 (0–4)	1 (0–7)	1(0–5)	1 (0–4)	0.59
**Maximum BedsidePEWS**, median (IQR)	10 (8–12)	9 (7–11)	9 (7–10)	7 (7–8)	10(8–13)	9 (8–11)	**0.02**
**Chronic Disease,** n (%)	23 (62)	8 (62)	9 (53)	22 (69)	62(63)	127 (98)	** < 0.0001**
**Diagnosis,** n (%)							** < 0.0001**
Haematology-Oncology,	7 (19)	2 (15)	5 (29)	10 (31)	24(24)	13(10)	
Cardiac	-	1 (7)	2 (12)	9 (28)	12(9)	12 (12)	
Respiratory	18 (49)	7 (54)	9 (53)	11 (34)	45(45)	50 (39)	
Neurological or Endocrine	12 (3)	3 (23)	1(6)	2 (6)	18(18)	12 (9)	
**CRIS**							** < 0.0001**
(1–4) (Early) n (%)	35 (95)	11 (85)	14 (82)	26 (81)	86 (87)	129	
> = 5 (Late) n (%)	2 (5)	2 (15)	3 (18)	6 (19)	13 (13)	0	
Median (IQR)	1 (1–2)	1 (1–2)	1 (1–2)	1(1–3.5)	1 (1–3)	1(1–1)	** < 0.0001**
**Recent transfer, n (%)**	21 (57)	5 (38)	7 (41)	4 (13)	37(54)	31(46)	**0.03**
ER < 24 h	13 (35)	2 (15)	4 (24)	1 (3)			
PICU readmissions < 48 h	4 (11)	-	1 (6)	1 (3)			
Other	4 (11)	2 (15)	2 (12)	2 (6)			
**PICU consult** n (%)	30 (81)	13 (100)	13 (76)	27 (84)	83(84)		** < 0.0001**
**Intubation** n (%)	1 (3)	0	0	5 (16)	6 (6)	0	**0.005**
**First EI score**, median (IQR)	6(4–7)	5(4–6)	4(3–5)	4(2–4)	5(4–6)	2(4–4)	** < 0.001**
**Ward Physician review** < 2–4 h, n (%)	29 (78)	11(84)	12(71)	19(59)	71(72)	71(55)	**0.01**
**PICU consult** < 6 h from first BedsidePEWS ≥ 7, n (%)	30 (81)	8(61)	8(47)	10(31)	56(57)	13(10)	** < 0.0001**
**BedsidePEWS documentation** hourly, n (%)	19(62)	5(38)	3(19)	3(9)	30(32)	17(13)	**0.001**
**ECG + SpO2**, n (%)	27(73)	12(92)	16(100)	32(100)	87(88)	108()	0.28
**Escalation index** (median, IQR)	4 (2–4)	3(2–4)	4(3–5)	4(2–6)	6(5–7)	4(3–5)	** < 0.0001**
**Q: Would you have called for an urgent PICU consult for this patient**							
Phisicians, median (IQR)	10(10–10)	10(10–10)	10(9–10)	10(8–10)	10(9–10)	5(2–8)	** < 0.0001**
Nurses, median (IQR)	9(9–10)	10(10–10)	10(10–10)	9(6–10)	10(9–10)	8.5(4–10)	**0.002**
**Q: How much would you have been surprised if this patient arrested during your shift**							
Phisicians, median (IQR)	6 (3.5–7)	3(2–5)	5(3–9)	6(3–9)	5(2–7)	7(3–9)	**0.01**
Nurses, median (IQR)	4(2–6.5)	4(2–7)	4(2–7)	4(2–7)	4(2–7)	3.5(2–6)	0.59

Legend: *EI* Escalation Index, *CRIS* Childrens’ Resuscitation Intensity Scale, *ER* Emergency Room, *PICU* Pediatric Intensive Care Unit, *ECG* Electrocardiogram, *SpO2* Peripheral Oxygen Saturation.

PICU admission more than 24 h after enrolment occurred in 21 (29%) patients at a median (IQR) of 18 days (7–34) following ward admission. There were no PICU admissions within 48 h of enrolment, suggesting that the use of 24-h disposition ensure an appropriate separation of the patients into the ward and ICU groups. Patients admitted to the ICU after 24 h of enrolment had a cardiac diagnosis (*n* = 18, 86%), one resuscitation team call was made, and all were admitted with CRIS scores equal to 4 or less

^a^duration of the time interval

**P value = PICU admission (YES) vs no PICU admission (NO)

Patients admitted to PICU had BedsidePEWS scores that were either high at enrolment or rose after the initial 6-h block (Fig. 3), 83 (84%) had PICU consults made to facilitate care, with a median (IQR) CRIS score of 1 (1-3). There were 13 (13%) late PICU admissions that included 5 resuscitation team calls, of which 4 (4%) patients were intubated prior to PICU admission. There were no deaths. Children who remained in the inpatient wards at 24 h had more escalation of care if their initial BedsidePEWS scores were higher; overall BedsidePEWS scores that became lower over time, had lower levels of escalation – suggesting de-escalation—over the 24 h (Fig. 3) and had physicians rating their concern higher in patients for whom there was greater escalation (Table 5).

**Table 5:** Patient characteristics and staff perceptions of clinical deterioration, by ICU admission and escalation of care^a^

	**PICU admission “24 h**				
	**NO**		**YES**		*P* value*
**Maximum Escalation Index score**	< 5	≥5	< 5	≥5	
**Patient characteristics**					
Maximum BedsidePEWS in last full 6- hour period, median (IQR)	7 (6–9)	7 (6–8)	9 (7–14)	10 (8–12)	0.37
Maximum BedsidePEWS median (IQR)	9 (7–11)	11 (9–11)	10 (8–14)	10 (8–12)	**0.02**
Maximum BedsidePEWS ≥10, n (%)	46/113 (41)	11/16 (69)	21/40 (53)	34/59 (58)	0.142
CRIS, n (%)					
1–4	113 (100)	16 (100)	33 (83)	53 (90)	** < 0.0001**
5–7	0 (0)	0 (0)	7 (17)	6 (10)	
Recent transfer, n (%)					
Yes	25	6	12	25	**0.03**
PICU Team Called, n (%)					
< = 6 h	4 (4)	9 (56)	17 (42)	39 (66)	** < 0.0001**
6–12 h	1 (1)	3 (19)	2 (5)	7 (11)	
> 12 h	0	3 (19)	5 (12)	13 (22)	
no call	108 (96)	1 (6)	16 (40)	0	
Medication complexity, n (%)					
Yes	72 (64)	6 (37)	21 (53)	31 (53)	**0.230**
No	41 (36)	10 (63)	19 (47)	28 (47)	
Isolation, n (%)					
Yes	34 (31)	5 (33)	12 (30)	27 (46)	**0.21**
No	76 (69)	10 (67)	28 (70)	32 (54)	
Chronic disease, n (%)					
Yes No	112 (99) 1 (1)	15 (94) 1 (6)	26 (65) 14 (35)	36 (61) 23 (39)	** < 0.0001**
HCP perception of patient deterioration, mean, SD					
Q: How much would ou have been surprised if this patient arrested? Physicians	4.85 ± 3.43	7.86 ± 2.19	8.53 ± 3.11	9.61 ± 0.79	**0.02**
Q: Would you have called for a PICU consult fo this patient? Physicians	4.54 ± 3.40	5.17 ± 2.64	8.60 ± 3.11	9.46 ± 0.88	** < 0.0001**
Q: How much would you have been surprised if this patient arrested? Nurses	6.88 ± 3.38	8.67 ± 1.86	8.22 ± 2.97	9.06 ± 1.98	0.75
Q: Would you have called for a PICU consult for this patient? Nurses	6.40 ± 3.20	6.83 ± 2.56	8.57 ± 2.69	9.09 ± 1.99	**0.002**

Patients who were not admitted to the PICU, with higher BedsidePEWS scores and Escalation Indexes had the greatest improvement (lower BedsidePEWS scores) at the end of the observation period. PICU = Pediatric Intensive Care Unit;  CRIS = Children’s Resuscitation Intensity Score; HCP = Health Care Professionals; Q = Question

^a^This table compares escalation practices (the highest escalation index) in patients admitted to PICU and patients who remained on the wards during the 24-h observation period. There was a median (IQR) of 2 (1-3) 6-h periods with one or more BedsidePEWS scores ≥ 7 in patients admitted to the PICU and 3 (3-4) in patients remaining on the ward. These permitted the calculation of 574 Escalation Index values. The escalation index ranges from 1–7. Escalation of care was classified as “low escalation” for an escalation index score = 1–4, and “high escalation” for an escalation index score = 5–7

^*^P = PICU admission vs no PICU admission

## Discussion

The aim of this study was to describe escalation of care and the occurrence and timing of PICU admission in a cohort of pediatric inpatients with acute worsening of their clinical condition. A prospective evaluation of 228 patient-episodes of increased severity of illness for at least four hours found that escalation of care over 24-h varied with patient characteristics and ongoing severity of illness. This is the first study that shows differences in escalation trends among children admitted to hospital wards, whereby children with acute conditions without baseline chronic conditions or children in need of isolation due to immune deficiencies or infections are more likely to be receiving earlier increased attention from the ward team when their BedsidePEWS score is ≥7. Greater escalation occurred also in children rated by healthcare professionals with a higher risk of deterioration and higher need for a PICU consult. This study also reported on the timeliness and magnitude of escalation in a children’s hospital, which are essential determinants of earlier PICU admissions and prevention of critical illness.

The four main findings relate to the relationship between severity of illness and escalation, and the timeliness of transfer from the ward. First, the observed correlation between the BedsidePEWS score and the EI may reflect the application of the BedsidePEWS score matched care recommendations. Considerable variability in the extent of escalation across the domains of vital signs monitoring, documentation, secondary reviews by ward based clinicians, and PICU consultations demonstrate the application of clinical judgment by involved clinicians, potentially reflecting the consideration of the score in the broader contexts of patients, ward environment, and temporal trends [9, 10]. The patients who remained on the ward for 24 h after enrolment had decreasing BedsidePEWS scores and relative de-escalation.

Second, healthcare professionals’ perceptions of patient’s risk were linked to escalation decision making about monitoring, secondary nursing and medical reviews, consultations with the PICU for management recommendations and consideration of PICU admission. This finding shows the responsiveness of the EI to HCPs’ clinical judgement of critical deterioration in children. A lower association might have been found if nurses had been blinded to patient disposition, considering a potential risk of recall bias.

Third, among patient and organizational factors, isolation was associated with escalation of care while having a chronic disease was inversely related. We noted that in 127 of the 129 episodes for which patients remained on the wards, involved patients with chronic diseases, and that the patients receiving care in isolation rooms were also more likely to receive more attention. Chronic disease was an important modifier of the extent of escalation—patients with chronic diseases were less likely to have care escalated. This may reflect a greater understanding of the basis of the patients’ physiological conditions, of anticipated trajectories, of higher ‘baseline’ scores, preferences to keep patients within specialized wards or other unmeasured factors. On the other hand, almost all patients with isolated acute severe illness were admitted to the PICU. Other factors including age, diagnosis, and recent transitions from the ICU and Emergency department were not significantly associated with escalation of care in multi-variable analysis.

Fourth, timing of escalation is essential to prevent or determine earlier PICU admissions to reduce severity of illness. This study showed an increasing escalation trend starting 12 h after enrollment and 12 h before PICU admission, from a partial escalation upon enrollment, showed by an intermediate EI value. Moreover, most children requiring advanced airway management in this study received prolonged observation on the ward up to 24 h, suggesting a late admission to the PICU. The role of the PICU team in facilitating PICU admission warrants further consideration. Increased risk of PICU admissions and mortality have been reported for patients who manifested prolonged clinical deterioration on the wards, suggesting the relevance of timely involvement of PICU teams in their management.

In addition, the cohort of children at high risk who stayed on the wards were almost all with chronic conditions. The one third that had a PICU urgent admission in a median (IQR) of 17.5 (6.5–32) days after enrolment, suggests ongoing elevated risk and raises questions about the ideal threshold for ICU admission [11, 12]. Normalizing the score of patients with complex and chronic illnesses can cause the underestimation of these children’s risk potentially leading to unexpected critical events [13]. A ‘score to PICU door’ time to prevent delays in recognition and treatments of deteriorating children might be taken into consideration to define safer care in those children [14].

### Limitations

This study has some limitations. First, since this was a monocentric study, the generalizability of the findings to hospitals with different escalation practices and response systems to critically-ill children may be limited. Second, other factors may explain decisions to admit to PICU – for example monitoring for arrhythmia, for hemorrhage, for trending the lactate, or closer observation of electrolytes, or clinical preference. Other measures such as laboratory, radiologic, diagnostic tests or other interventions almost certainly influenced clinical decision making, and should be reflected in the clinicians’ assessment of risk of cardiac arrest and their perception of the benefit of a PICU consultation. Future work may consider calibrating the elements of the EI, to confirm the weight of each element within the score [15].

Third, in this study we were unable to assess the effect of the BedsidePEWS escalation algorithm on important patient outcomes, such as mortality, mechanical ventilation days or PICU length of stay. Greater adherence to the SMCR may have prevented late PICU urgent admissions but whether this is associated with an overall reduction of PICU urgent admissions needs to be further explored.

Fourth, we examined patients with BedsidePEWS ≥7, excluding children with lower scores. Thus, we were not aware of the pattern of escalation of care and the characteristics of patients urgently transferred to PICU with BedsidePEWS scores < 7.

Lastly, the retrospective nature of HCPs’ questionnaires possibly increased the risk of recall bias. However, the questionnaires were anonymous, thus limiting the risk of any bias related to social desirability.

## Conclusion

The evaluation of a cohort of children with acutely increased severity of illness showed variability in escalation responses, which was related to temporal changes in the severity of illness, background patient and environmental factors. EI scores were higher in children urgently admitted to PICU than in children who remained in the wards, for whom EI scores gradually decreased. Bedside PEWS and isolation were associated with escalation of care, while chronic illness was inversely related. Use of measures such as the escalation index can help to adapt responses to evolving critical illness as part of the assessment of the effectiveness of rapid response systems.

## Supplementary Information

**Additional file 1.** Supplementary file 1.

**Additional file 2: Supplementary Electronic Table 1.** Escalation of care at enrollment.

## Data Availability

The datasets used and analyzed during the current study are available from the corresponding author following a justified request.

## Abbreviations

BedsidePEWS: Bedside Pediatric Early Warning System
CRIS: Children Resuscitation Intensity Score
EI: Escalation Index
HCP: Health Care Provider
ICU: Intensive Care Unit
PICU: Pediatric Intensive Care Unit
SMCR: Score Matched Care Recommendations
