# Supplementary material for: Escalation of care in children at high risk of clinical deterioration in a tertiary care children’s hospital using the Bedside Pediatric Early Warning System
Source: BMC Pediatr. 2022 Sep 7;22:530. doi: 10.1186/s12887-022-03555-0 (PMC9450425; doi:10.1186/s12887-022-03555-0)
Supplement: Supplementary file 1 — Additional file 1. Supplementary file 1. [file 12887_2022_3555_MOESM1_ESM.docx]

Supplemetary file 1

The CRIS, ranging from 1-7, is categorized into 2 groups to describe early and late PICU admission: 1) scores 1-4, low-medium intensity of care, including one of the following interventions: positive pressure ventilation, intubation <12 hours before transfer, intubation <1 hour after transfer, inotropes, vasoactive therapy or fluid boluses of 60 ml/Kg or more in 12-hours before ICU; 2) scores 5-7, high intensity of care, including more than one of the previous interventions, cardiopulmonary resuscitation or death [9].
