# Supplementary material for: Escalation of care in children at high risk of clinical deterioration in a tertiary care children’s hospital using the Bedside Pediatric Early Warning System
Source: BMC Pediatr. 2022 Sep 7;22:530. doi: 10.1186/s12887-022-03555-0 (PMC9450425; doi:10.1186/s12887-022-03555-0)
Supplement: Supplementary file 2 — Additional file 2: Supplementary Electronic Table 1. Escalation of care at enrollment. [file 12887_2022_3555_MOESM2_ESM.docx]

**Supplementary Electronic Table 1**: Escalation of care at enrollment

|  |  | **Total** | | **PICU urgent admissions** | | **Ward patients** | **P-value** | |
| --- | --- | --- | --- | --- | --- | --- | --- | --- |
| N |  | | 228 | 99 | 129 | | |  |
| Vital signs and BPEWS scoring*, N (%) |  | |  |  |  | | | <0.001 |
| <1 hours |  | | 47 (21) | 30 (30) | 17 (13) | | |  |
| 1-4 hours |  | | 165 (73) | 56 (57) | 109 (85) | | |  |
| >4 hours |  | | 69 (4) | 6 (6) | 3 (2) | | |  |
| Not applicable |  | | 5 (2) | 5 (5) | - | | |  |
| Type of monitoring, N (%) |  | |  |  |  | | | 0.463 |
| Intermittent |  | | 17 (7) | 5 (5) | 12 (9) | | |  |
| Continuous SpO2 |  | | 15 (7) | 6 (6) | 9 (7) | | |  |
| ECG Monitor + SpO2 |  | | 195 (86) | 87 (89) | 108 (84) | | |  |
| Physician reviews |  | |  |  |  | | |  |
| Number of Physician review in 24h (n), mean±SD |  | | 3.11±2.84 | 3.86±2.38 | 2.55±1.75 | | | <0.001 |
| Physician review (timing) N (%) |  | |  |  |  | | | 0.093 |
| ≤ 4 hours |  | | 142 (62) | 71 (72) | 71 (55) | | |  |
| 4- 6 hours |  | | 11 (5) | 3 (3) | 8 (6) | | |  |
| 7-12 hours |  | | 37 (16) | 10 (10) | 27 (21) | | |  |
| > 12 hours |  | | 18 (8) | 7 (7) | 11 (9) | | |  |
| No call |  | | 20 (9) | 8 (8) | 12 (9) | | |  |
| MET/RRT review, N (%) |  | |  |  |  | | | <0.001 |
| ≤ 6 hours |  | | 69 (30) | 56 (57) | 13 (10) | | |  |
| ≤ 12 hours |  | | 13 (6) | 9 (9) | 4 (3) | | |  |
| > 12 hours |  | | 21 (9) | 18 (18) | 3 (2) | | |  |
| No calls |  | | 125 (55) | 16 (16) | 109 (85) | | |  |

Note: *The Escalation Index was calculated at the 1st BPEWS>=7
